# Sn/Pb ratio variation in spherical structures deposited on silicon surface using plasma focus

**DOI:** 10.1016/j.heliyon.2023.e17098

**Published:** 2023-06-12

**Authors:** M. Ahmad, M. Akel, Sh. Al-Hawat

**Affiliations:** a IBA Laboratory, Physics Department, Atomic Energy Commission of Syria, P.O. Box: 6091, Damascus, Syria; b PF Laboratory, Physics Department, Atomic Energy Commission of Syria, P.O. Box: 6091, Damascus, Syria

**Keywords:** Plasma focus, XPS, SEM, Tin, Lead

## Abstract

The deposition of Sn and Pb elements on top of Si surface was realized using plasma focus device. Due to the special characteristic of this type of plasma, the silicon substrate is heated by the bombardment of plasma ions, before the deposition of these elements sputtered from the anode. The deposition of the two elements was found to be influenced by substrate-anode distance as a consequence of surface heating. It was found that the relative amounts between the two deposited elements was not the same as their original ratio in the anode before sputtering. The ratio between Sn and Pb varies with increasing depth into the SnPb deposited on the Si substrate. Additionally, the size of micro spherical structures formed on the surface affected the ratio of the two deposited elements. The variation of the ratio is explained as result of deposition/evaporation competition influenced by the surface heating.

## 1 Introduction

In the past decade, many research works have been devoted to the applications of plasma focus device (PF) in surface material science, mainly, ion implantation [[1], [2], [3], [4], [5]], structural modification [[6], [7], [8]] and thin films deposition [7,9,10]. However, the process that involves the deposition of elements sputtered from the anode on top of the substrate needs more investigation to both understand and perform controlled film deposition. In general, PF device is used to deposit layers composed of the anode materials. The deposition process involves generation of both ions and electrons that are accelerated in opposite directions. The plasma ions go in the direction of the substrate while electrons heat the top of the anode leading to sputtering and evaporation of anode materials. The sputtered particles spread in all directions away from the top surface of the anode, where most of them go behind the plasma ions towards the substrate. The substrate surface is heated up by the plasma ions bombardment before the arrival of the sputtered anode materials. It is noteworthy to mention that the substrate surface can be melted [6] depending on the distance between the substrate and the anode. The thermal relaxation takes place in few microseconds after the end of the ion beam arrival [11]. The sputtered materials from the anode, caused by the electrons bombardment, arrive to the heated surface substrate during and after the thermal relaxation. At a close distance from the anode, the sputtered materials from the anode undergo shorter delay to reach the heated surface. Therefore, according to the thermal relaxation stage of the substrate surface [11], the formation of layer from the sputtered elements on the Si surface will go through deposition, evaporation and sublimation. Such process involving surface heating by plasma ions before the deposition of anode elements influences the surface structure and the size of deposited particulates [7]. The sputtering of different elements from the anode by plasma electrons can lead to have these elements of different stoichiometry in the vapor phase before its deposition on a substrate. Nevertheless, the temperature of the substrate heated by the ions plasma can also influence the stoichiometry of elements during and after deposition.

In this work, the PF was used to deposit two elements (Sn and Pb). The choice of these elements was not only for its importance in structural, optical and electrical applications [[12], [13], [14], [15], [16], [17]] but essentially, it was adopted for its low melting points (231.9 °C for Sn and 327.5 °C for Pb) in order to allow understanding the deposition/evaporation processes of the two elements on the top of Si substrates which are heated by ion plasma at different temperatures, according to the anode-substrate distance. It is worth mentioning that the use of high melting point elements in the anode (as Molybdenum) will lead to heating the substrate without deposition of Molybdenum [1]. Additionally, the chemical state (metallic or oxide) of the deposited Sn and Pb elements were analyzed using the XPS–depth profiling. This could be important in the field of optical and electrical properties of the formed nano and micro structures that can be formed using PF [7] on the silicon surface.

## 2 Experimental details

The low energy (2.8 kJ) Mather-type plasma focus device described in Refs. [1,6,7] was used in this work. It was operated under N_2_ gas at pressures of 0.8 mbar. The top of the central anode was encapsulated with SnPb alloy at atomic stoichiometry of 60% Sn and 40% Pb. These two elements were sputtered and then deposited on silicon substrate. The silicon substrates of 1 × 1 cm^2^ areas were positioned at different distances from the anode in order to both determine the amount of deposits and investigate the effect of surface heat by plasma ions [1]. The elemental composition of the deposited structure on the silicon surfaces was studied by X-ray photoelectron spectroscopy technique (XPS). More detail of the XPS experimental setup and analysis are described in previous work [7]. Where, the charging produced during the XPS measurements is overcome by using electron gun to neutralize the sample surface charge. The binding energies in the spectra were referenced to the carbon, C 1s peak at 284.5 eV. The XPS analysis was performed in order to trace the deposited Sn and Pb on top of the surface of silicon substrates, which were heated at different temperatures by plasma ions, by positioning the substrates at different distances from anode [11]. Depth profiling-XPS analysis was performed in order to trace in depth the composition and chemical state of the formed structure on the silicon substrate. This is realized by etching the treated silicon surface using ions source operated with argon. The ion beam incidence angle and energy were 45° and 5 keV, respectively. The crystalline structure of the deposited tin and lead was characterized using X-ray diffraction (θ–2θ XRD) with the Cu K_α_ radiation. An additional experiment by sputtering pure Sn from the anode is realized in order to compare the crystalline structure formed on the silicon surface with the case of sputtering Sn and Pb together. A scanning electron microscope (SEM) (from TESCAN VIGA II XMU, Brno, Czech Republic) was employed to examine the surface morphology induced on the silicon substrate s. Additionally, the energy dispersive X-ray technique (EDX) equipped with the SEM microscope was used to enable the determination of the surface composition in selected structures and areas.

## 3 Results and discussions

**Fig. 1:**
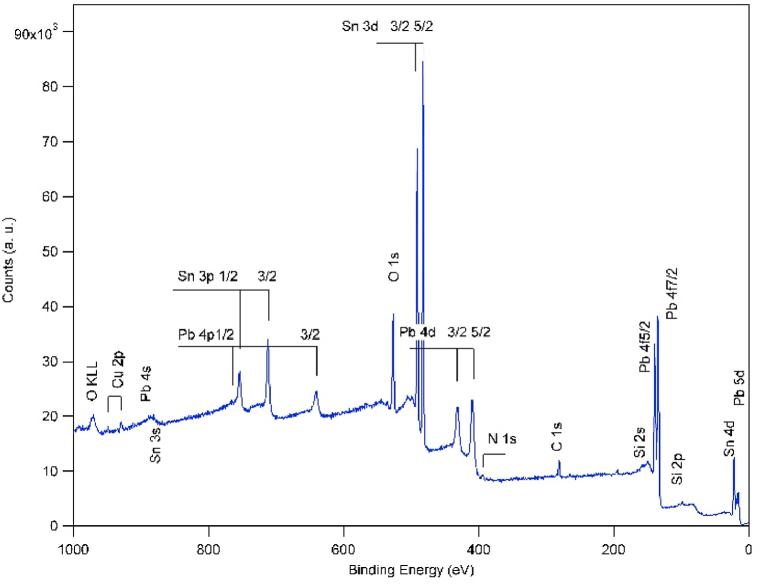
XPS survey spectrum of Si substrate treated by 10 shots of N_2_ PF plasma at 3 cm from the SnPb anode.

Fig. 1 shows XPS survey spectrum taken from the surface of Si sample treated by 10 shots of N_2_ plasma focus at a distance of 3 cm from the Sn–Pb anode. As expected, the spectrum shows the presence of both elements sputtered from the anode material and deposited on top of the silicon substrate. These two elements present on the top of the anode with atomic concentration of 60% and 40% for Sn and Pb, respectively were found in different stoichiometry on the silicon substrate. Table 1 presents the elemental concentrations obtained from the XPS spectra at different etching time (depth) of the sample presented in Fig. 1. The minor presence of chlorine is due to contamination from previous experiment in the chamber. While the presence of silicon is due to the substrate that is not completely covered by the deposited elements as a continues layer which can be seen in Fig. 8 (below).

**Table 1:** Atomic concentration (%) of elements analyzed from XPS spectra in Fig. 1.

Etching time (min)	Sn	Pb	Si	N	Cu	O	C	Cl	Sn/Pb ratio
0	7.1	11.4	4.3	2.6	0.3	25.6	47	1.7	0.6
25	20.7	17.3	6.7	3.2	1.1	31.7	17.4	2.0	1.2
50	24.5	18.0	8.3	1.0	0.7	34.0	12.2	1.4	1.4

The ratio between these two elements is far from what it is expected (Sn/Pb = 1.5). It is found that this ratio varies according to the depth from the surface of the deposited materials. The relative amount of Sn/Pb decreases near the surface due to the evaporation or sublimation from the heated surface of silicon substrate, which is exposed to heat by the plasma ions [11].

**Fig. 2:**
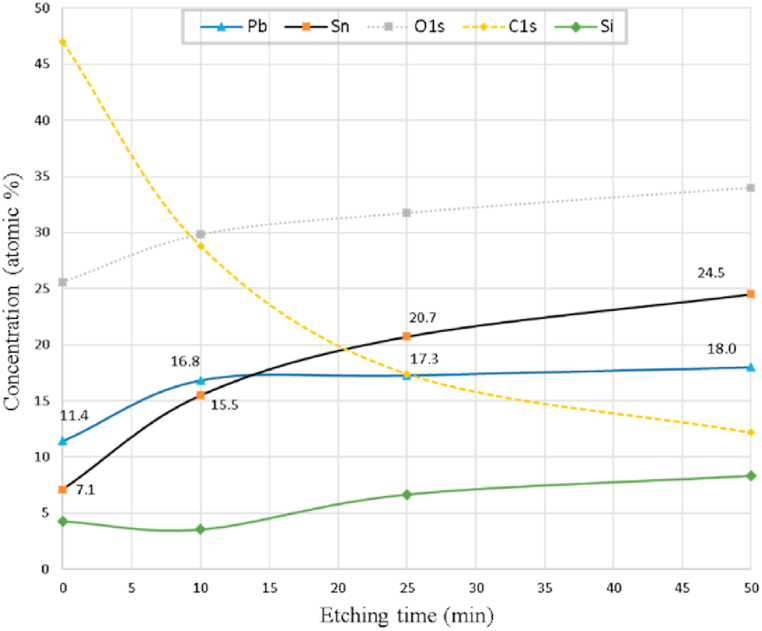
XPS depth profiling showing the variation of elemental concentration when increasing depth on the silicon substrate treated at 3 cm from the anode by 10 shots of nitrogen plasma.

The depth profiling of elemental concentration (Fig. 2) shows an enrichment of Pb at the top surface which is exposed to the evaporation or sublimation more than in depth. This is in good agreement with [[18], [19], [20], [21]], who found an enrichment of Pb in lead-tin alloy subject to either surface heat or ion bombardment. This enrichment was explained by Refs. [22,23] as follows: heating the surface of an alloy in vacuum will lead to enrichment with component of the alloy that has the lowest heat of sublimation. The heat of sublimation of Sn is higher than that of Pb [[23], [24], [25]].

**Fig. 3:**
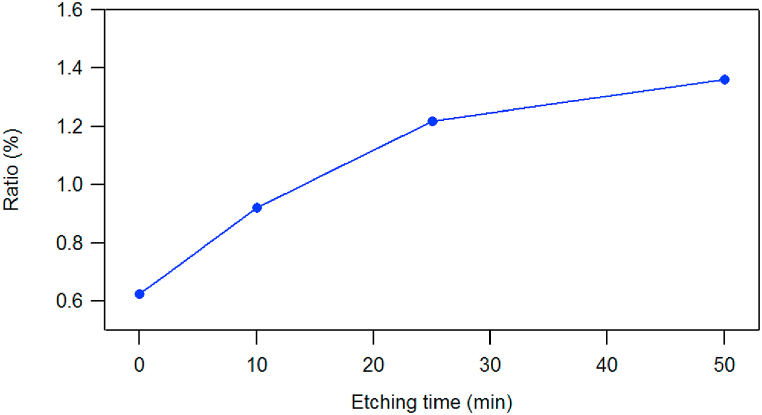
The ratio of Sn/Pb as increasing depth on the treated silicon surface by 10 shots of nitrogen plasma at 3 cm from anode.

**Fig. 4:**
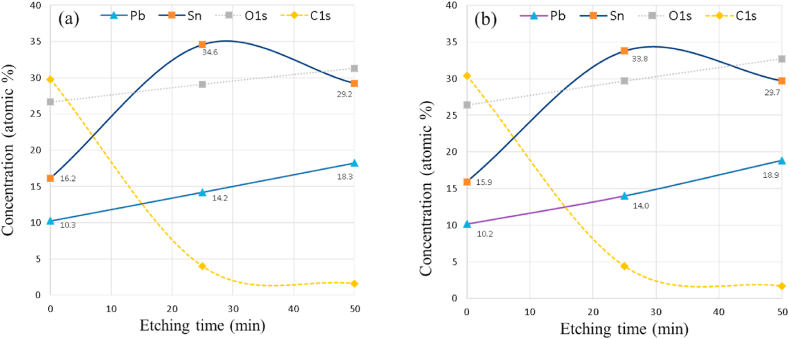
XPS depth profiling showing the variation of elemental concentration with increasing depth on the surface of the treated silicon substrate by 10 shots of nitrogen plasma at 5 cm (a) and 7 cm (b) distances from the anode.

The Sn/Pb ratio at different depths is presented in Fig. 3. It shows that this ratio increases with depth in sample prepared at the closest distance from the anode (3 cm). However, the depth profiling of samples treated by plasma shots at farther distance, i.e. more than 3 cm from the anode, show an enrichment of Sn in a subsurface layer (Fig. 4a and b); this enrichment of Sn is not observed in the samples treated at 3 cm (Fig. 2). The reason could be attributed to the difference in the surface heat that is more elevated at the closest distances [11] therefore, only the top surface lose more Sn by evaporation or sublimation at the farthest distance from the anode.

**Fig. 5:**
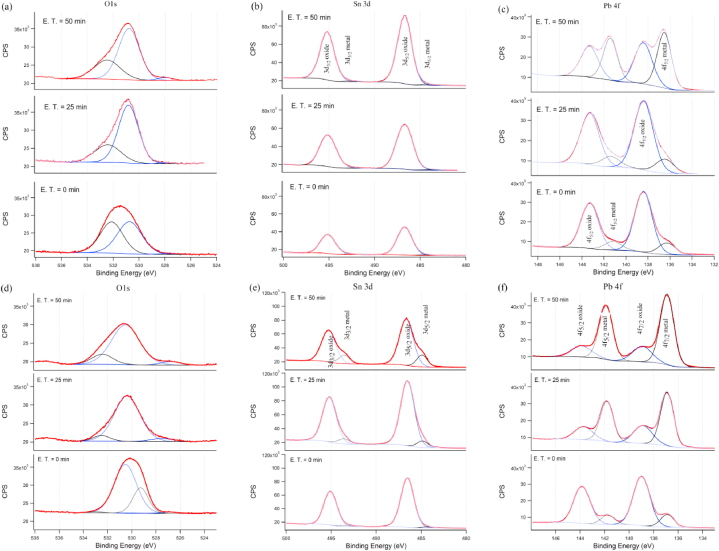
High resolution XPS at three different etching time (E. T.) for O1s, Sn3d and Pb4f, taken from sample treated by PF, at 3 cm from anode (a), (b) and (c), respectively and at 7 cm from anode (d), (e) and (f), respectively.

The high resolution XPS spectra in Fig. 5 show the splitting of Sn 3d into two sub-bands: Sn3d_3/2_ and Sn 3d_5/2_ (Fig. 5b and e) and the splitting of Pb 4f into two sub-bands: Pb 4f_7/2_ and Pb 4f_5/2_. (Fig. 5c and f). The fitting revealed the presence of metallic and oxide forms of both elements. The metallic Sn 3d_3/2_ and Sn 3d_5/2_ are found at binding energies of 493.5 eV and 484.9 eV, respectively; while the oxides are found at 495.3 eV and 486.7 eV, respectively. The spectra resolution is not optimized to distinguish the degree of oxidation of Sn^+4^ and Sn^+2^ in the oxide form of tin as in Refs. [[26], [27], [28]]. The binding energies of Pb 4f sub-bond (Pb 4f_7/2_ and Pb 4f_5/2_) in metallic form are found at 136.9 eV and 141.9 eV, respectively; they are shifted in the oxide form to 138.9 eV and 143.9 eV, respectively. These binding energies of lead oxide are in accordance with reference [29], that showed that the shift was dependent on the temperature.

**Fig. 6:**
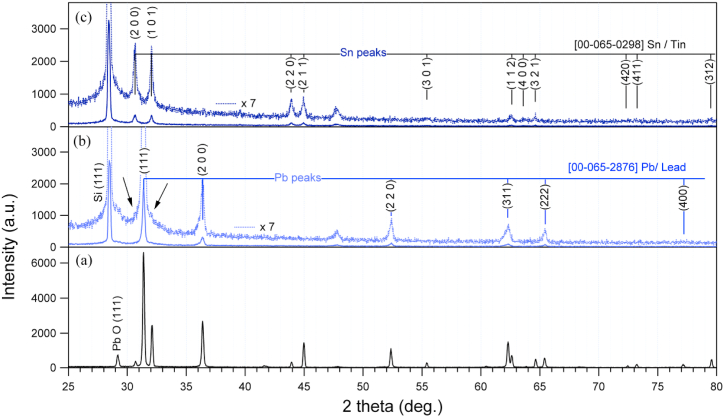
θ–2θ XRD patterns taken from the encapsulated Sn–Pb of the top of the anode (a). The presented patterns in (b) and (c) are from the Si samples prepared by PF plasma using Sn–Pb and Pure-Sn in the anode, respectively. The indices are referred to the structure of metallic Lead and Tin.

The variation of metallic and oxide forms observed in the spectra presented in Fig. 5 indicates that the tin in the upper surface (with no etching) is present in the form of the oxide while lead is present in both forms; oxide and metal. The metallic forms of both elements increase with increasing depth while oxide forms decrease. The oxide form of Pb decreases from 83% at the top surface to 23.4% in 50 min of etching time (see Table 2). Meanwhile, SnO_2_ decreases from 97% to 82.9%. The reduction of lead oxide by ion bombardment could be the reason for increasing metallic lead [30]. However, the presence of lead in crystalline metallic form, observed by XRD (Fig. 6) is more likely due to that lead is deposited in metallic form and not due to reduction by sputtering effect during the etching. Considering that XRD probes deeper than XPS, the metallic form is present clearly due to the deposition process. Fig. 6 shows three different XRD spectra demonstrating that metallic form of Sn is observed in the sample that has been prepared using pure Sn on the top of anode (Fig. 6c) and in the (Sn–Pb) alloy used on the top of anode (Fig. 6a). This means that when Sn is deposited with Pb, the crystalline form of metallic Sn is minor (indicated by arrow in Fig. 6b) and the major amount of metallic Sn is deposited as amorphous form. Nevertheless, the higher amount of Sn is not a metallic from. It is highly oxidized (as seen by XPS in Fig. 5b and e) in the amorphous form as there is no signal of tin oxide in the XRD spectrum. Both Fig. 5 and Table 2 show that within the investigated depths there is more oxidation occurred in the sample prepared at the closest distance from the anode (3 cm illustrated in Fig. 5a, b and c) compared with the one at the farthest distance (7 cm illustrated in Fig. 5d, e and f). This could be due to the exposure of surface to high temperature by the ion bombardment of surface from plasma ions at the closest distance from the anode. Another remark in Fig. 5 is that the metallic form of Sn is scarcely detectable within the investigated depth in the sample treated at 3 cm, as seen for the metallic Pb (Fig. 5a and b). While metallic Sn is closer to the surface in the sample treated at 7 cm (Fig. 5e).

**Table 2:** Percentages of metallic and oxide forms of Pb and Sn obtained from fitting high resolution XPS spectra.

Sample	Etching time (min)	Pb (%)		Sn (%)	
		Oxide	Metal	Oxide	Metal
Sample at 3 cm	0	84.1	15.9	100	0
	25	81.8	18.2	99.2	0.8
	50	47.8	52.2	98.4	1.6
Sample at 7 cm	0	83.1	16.9	97	3
	25	31.9	68.1	91.8	8.2
	50	23.4	76.6	82.9	17.1

The fitting of oxygen (O 1s) peak shows different binding energies. One of them is found at 527.5 ± 0.6 eV. This could be attributed to lead oxide, as found by Ref. [31], where they found that O 1s at 527.7 eV is bonded to Pb in PbO_2_ and O 1s at 527.8 eV is bonded to Pb in PbO tetragonal). Also [32] found low binding energy of O 1s at 528.9 eV in PbO_2_. It is obvious from (Fig. 5a and d) that this oxygen at low binding energy is lead oxides. It could be attributed to mono or dioxide of Pb as in Refs. [31,32]. The main O 1s component found at 530.9 eV is corresponding to the binding energy of oxygen in SnO_2_ [33,34]. Other reference attributed this compound of O 1s at 530.9 eV to lead oxide (PbO_ads_) in adsorbed oxygen layer [35] or to PbO_rombic_ [36]. Another O 1s peak is found at binding energy of 532.4 ± 0.5 eV, the intensity of which increases with increasing depth. This peak can be attributed to O 1s in native SiO_2_ present on the surface before treatment by the PF. This attribution is supported by the same binding energy of (O 1s) in SiO_2_ by Refs. [[37], [38], [39]]. This peak of high binding energy component is attributed to the adsorbed oxygen in the form of OH groups at (532.2 eV) or water molecules at (533.2 eV), see Ref. [33] and references therein.

**Fig. 7:**
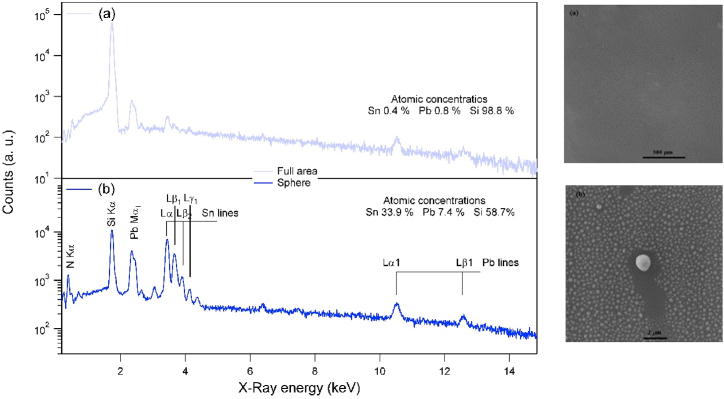
EDX spectra of sample treated by 10 N_2_ shots at 6 cm from the anode taken from full area 1900 × 1900 nm^2^ in (a) and from surface of 200 × 200 nm^2^ on a sphere of micro size (b) together with the corresponding SEM images.

**Fig. 8:**
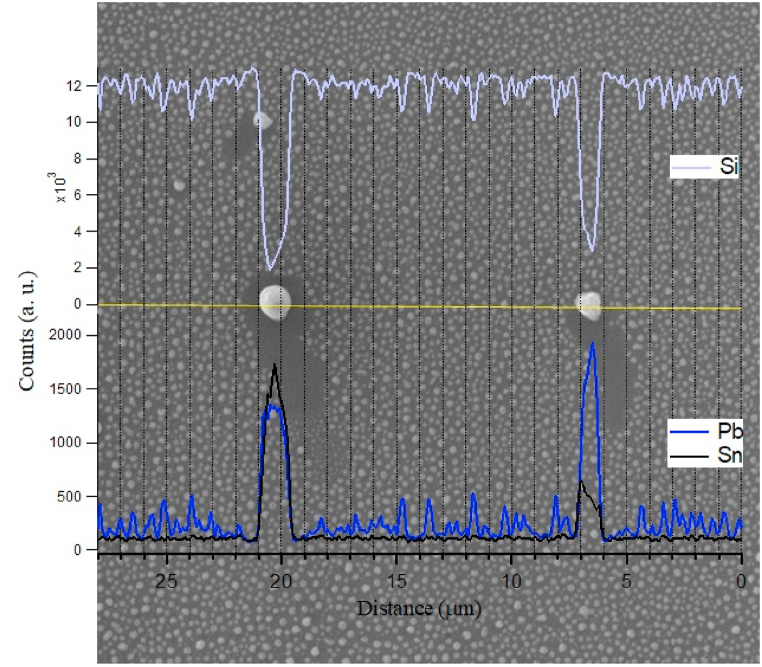
EDX line scan taken along two big microspheres on the surface of a sample treated by 10 N_2_ shots at 6 cm from the anode. This figure shows the dependence of Sn/Pb according to the spheres size.

XPS probes only few nanometers of the deposited structures, which have thicknesses of more than 100 nm (see SEM Fig. 7, Fig. 8). In these figures micro spherical structures are formed by the snow ball effect [7] where, large sphere can be formed by rooling over nano spheres agglomirating in bigger sphere. EDX together with SEM are used for tracing the presence of Sn and Pb elements in more depth of the substrate surface than XPS can do. In addition, it can help to investigate the composition of particulate structures, such as the spheres formed on the surface. Fig. 7 shows two EDX spectra taken from two places on the surface of sample treated by 10 nitrogen plasma shots at 6 cm from the anode. The spectrum taken from the sphere shows low signal from silicon compared to spectrum taken from large area. This could be attributed to the attenuation of the electrons before reaching the substrate through the micro sphere. The atomic concentration of Sn is higher than that of Pb in the spectrum taken from a micro sphere; while it is the opposite in the spectrum taken from a large area from the surface (1.9 × 1.9 μm^2^). This result is a consequence of the thickness of the sphere that can retain Sn, against the evaporation or sublimation, while the thinner hot surface loses Sn more than Pb. This finding support the results obtained by XPS analysis (Table 1). The line EDX scan of elements over two different size spheres presented in Fig. 8 shows the dependence of Sn/Pb ratio on the size of spheres where thicker sphere contains more Sn compared to Pb.

**Fig. 9:**
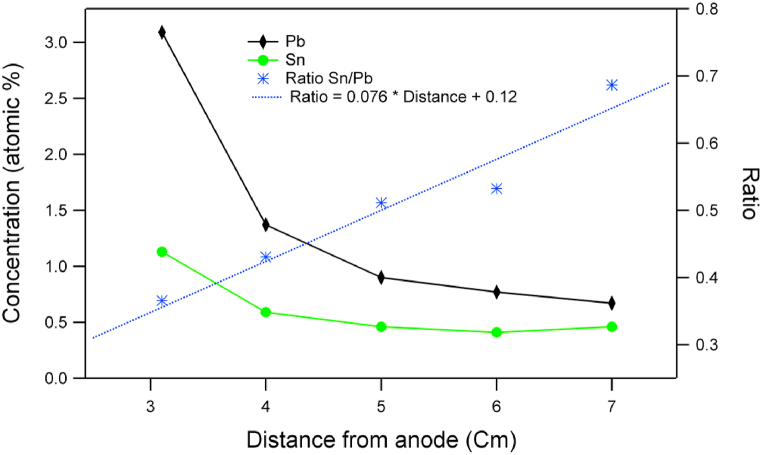
Atomic concentrations obtained from EDX measurements of samples prepared under 10 nitrogen shots at different distances from the anode.

The investigation of the atomic concentration of Sn and Pb from wide area on the surface of samples prepared under different distances from the anode shows decrease of the amounts of both elements (Fig. 9). However, there is an increase of the relative amount of Sn against Pb. This increase is due to the decrease of the surface temperature with increasing distance from the anode. Therefore, Sn can be more retained at the surface of the samples treated by PF at further distances than at closest distances from the anode.

## 4 Conclusions

This work demonstrates that plasma focus can be used for the deposition of two elements (Sn and Pb) onto silicon substrate. It was found that the deposited amount of Pb is higher than Sn, contrarily to its initial relative amount in the anode. Additionally, the distance from the anode, at which the samples are treated by the plasma shots, plays a key role in the amount and ratio of the two elements as a consequence of the surface heat sublimation or evaporation. The ratio of Sn/Pb is found to increase with increasing distance from the anode. This is due to the fact that the evaporation or sublimation have less effect at the furthest distance from the anode, where the silicon surface is less heated by plasma ions.

On the other hand, the XPS analysis of the treated Si surface reveals the increase of Sn with increasing etching (depth). Moreover, an enrichment of Sn was found at subsurface depth on the sample treated by plasma at the furthest distances from the anode. Such enrichment of Sn was also observed on the micro spherical structures formed on the Si surface. This can be explained as retention of Sn against the evaporation or sublimation in both, the thick structure (spheres) or from the deep regions of structures deposited on the silicon surface.

Furthermore, the non-oxidized metallic forms of deposited Sn and Pb elements are influenced by the surface heat, where more metallic forms are found at the higher depth of the deposited structures. Additionally, there are more metallic forms found at the surface of samples prepared at further distances from the anode.

Our experimental setup does not allow us to set anode-substrate distance above 7 cm, at which it is expected to have elements deposition ratio close to its initial stoichiometry in the anode.

## Author contribution statement

M. Ahmad; M. Akel; Sh. Al-Hawat: Conceived and designed the experiments; Performed the experiments; Analyzed and interpreted the data; Wrote the paper.

## Data availability statement

No data was used for the research described in the article.

## Declaration of competing interest

The authors declare that they have no known competing financial interests or personal relationships that could have appeared to influence the work reported in this paper.
